# Neurological and Neuropsychiatric Manifestations of Antiphospholipid-Antibody Syndrome (APS)

**DOI:** 10.7759/cureus.26022

**Published:** 2022-06-16

**Authors:** Saba Asif, Anoushka Bali, Ashujot Kaur Dang, Daniel A Gonzalez, Rajeswar Kumar

**Affiliations:** 1 Internal Medicine, Apollo Hospitals, Hyderabad, IND; 2 Research, Acharya Shri Chander College of Medical Sciences & Hospital, Jammu, IND; 3 Research, Government Medical College, Patiala, Patiala, IND; 4 General Physician, Universidad Catolica Santiago de Guayaquil, Guayaquil, ECU; 5 Medicine, Rajah Muthaiah Medical College and Hospital, Chidambaram, IND

**Keywords:** neuropsychiatric manifestations of aps, antiphospholipid antibody syndrome (aps), autoimmune disorder, psychosis, stroke in young

## Abstract

Antiphospholipid antibody syndrome (APS) is an autoimmune disorder mediated by the presence of a group of autoantibodies, specifically the anticardiolipin antibody (aCL), the beta-2 glycoprotein I (*β*2GPI), and the lupus anticoagulant (LA). Patients diagnosed with antiphospholipid antibody syndrome (APS) present with many symptoms, the most common being the consequence of thrombotic events that can be catastrophic and lead to mild to severe residual disabilities over a significant amount of time and can impair the quality of life. These events are often present in the younger population. Many times, these thrombotic events are heralded by a spectrum of psychiatric symptoms, which when worked up in the right direction may hint toward an oncoming thrombotic event and may potentially prevent those events by prompting primary prophylaxis treatment by the treating physician. In this review, we aim to comprehensively put forth the many neurological and neuropsychiatric manifestations of APS, their pathology, and management.

## Introduction and background

Antiphospholipid antibodies (aPLs) are a group of antibodies directed against phospholipid-binding plasma proteins. Antiphospholipid syndrome (APS) is an autoimmune disorder that requires the persistence of autoantibodies: anti-β2-glycoprotein I (anti-β2GPI), anticardiolipin (aCL), and lupus anticoagulant (LA) tested positive at least 12 weeks apart [1]. APS has been reported since the early 1980s in association with systemic lupus erythematosus (SLE). The actual incidence of APS in the United States is unknown. However, about 250,000 thrombotic events in the United States each year are linked to APS. In patients with associated SLE, LA titer predicts up to 50% of a 20-year risk of a thrombotic event, of which stroke is the most common [2]. The incidence of APS in the US is five in every 100,000 subjects/year, with a prevalence of 40-50 in every 100,000 subjects [3].

Clinical diagnosis of APS is often difficult and missed, not only because it is a rare disorder but also due to gaps in knowledge of its diagnosis and classification. APS is suspected in patients with recurrent early pregnancy loss, usually <10 weeks of conception, arterial or venous thrombosis with (secondary APS) or without SLE (primary APS), and in very rare cases, under a devastating event causing multiple thromboses involving more than two systems labeled catastrophic APS (CAPS) [2]. Currently, APS is classified based on the revised Sapporo classification of 2006 (Sydney criteria) as outlined in Table 1, but an international consensus to define, diagnose, and classify APS is ongoing [4]. According to Sydney criteria, the classification of APS requires at least one clinical (arterial/venous thrombosis or pregnancy loss) and one laboratory criterion [1].

**Table 1:** Revised Sapporo classification criteria for APS (2006). APS: antiphospholipid antibody syndrome.

Clinical criteria
Vascular thrombosis
≥1 Clinical episode of arterial, venous, or small vessel thrombosis in any tissue or organ.
Pregnancy morbidity
≥1 Unexplained death of a morphologically normal fetus at or beyond the 10th week of gestation.
≥1 Premature birth of a morphologically normal neonate before 34 weeks of gestation because of eclampsia, pre-eclampsia, or recognized placental insufficiency, or
≥3 Unexplained consecutive spontaneous abortions before the 10th week of gestation, with maternal anatomic or hormonal abnormalities and paternal and maternal chromosomal causes excluded.
Laboratory criteria
Lupus anticoagulant is present in plasma on ≥2 occasions at least 12 weeks apart.
Anticardiolipin antibody of IgG and/or IgM isotype, in medium or high titer (>40 GPL or MPL, or >99th percentile), on more than two occasions at least 12 weeks apart.
Anti-*β*2-glycoprotein I antibody of IgG and/or IgM isotype, in medium or high titer (>the 99th percentile), on ≥2 occasions, at least 12 weeks apart.

While recurrent pregnancy loss is the most common presentation of APS, a Euro-phospholipid group study involving 1000 patients done by Cervera et al. showed that neurological manifestations were often present and their prevalence was as listed below in Table 2 [5].

**Table 2:** Prevalence of neurological manifestations in a 1000 cohort study done by Cervera et al.

Symptom	Prevalence in APS
Transient ischemic attack (TIA)	11.10%
Ischemic stroke	19.8%
Cerebral venous thrombosis	0.7%
Seizures	7%
Headache	20.2%
Chorea	1.3%
Transverse myelitis	0.4%
Dementia	2.5%

Apart from the above-mentioned neuropsychiatric symptoms, case reports and case series highlighting manifestations of APS such as cognitive impairment, memory loss, acute psychosis, behavioral abnormalities, etc. have been published, whose incidence or prevalence is hard to define. These non-thrombotic, non-criteria neuropsychiatric manifestations rarely occur at presentation and are often misdiagnosed or diagnosed later when thrombotic events occur.

The pathogenesis of thrombotic events in APS is largely complex and involves several mechanisms that involve increased expression of procoagulant factors, endothelial cell damage, platelet, monocyte, and neutrophil involvement along with complement pathway activation [6]. Only animal models hypothesizing the pathogenesis of neuropsychiatric manifestations have been studied. The overall data from these animal models suggest immune-mediated pathogenesis with direct binding of aPL on neurons and glial cells, which occurs after disruption of the blood-brain barrier [7-9].

This review aims to summarize the neurological and neuropsychiatric manifestations of APS, the importance of early diagnosis and treatment, and whether or not anticoagulants in non-thrombotic disease impact the outcome of patients with APS.

## Review

Pathophysiology

Thrombotic Pathophysiology

**Figure 1:**
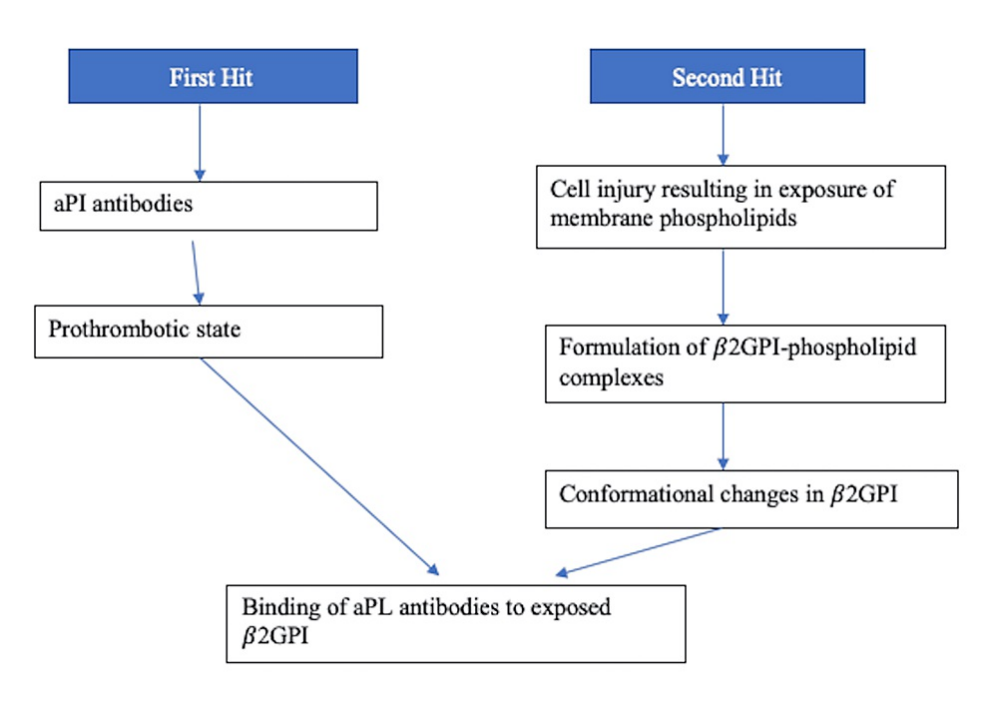
Depicting the two-hit hypothesis in the pathogenesis of APS. aPL: antiphospholipids, β2GPI: beta-2 glycoprotein I, APS: antiphospholipid syndrome, aPI: antiphospholipid.

**Figure 2:**
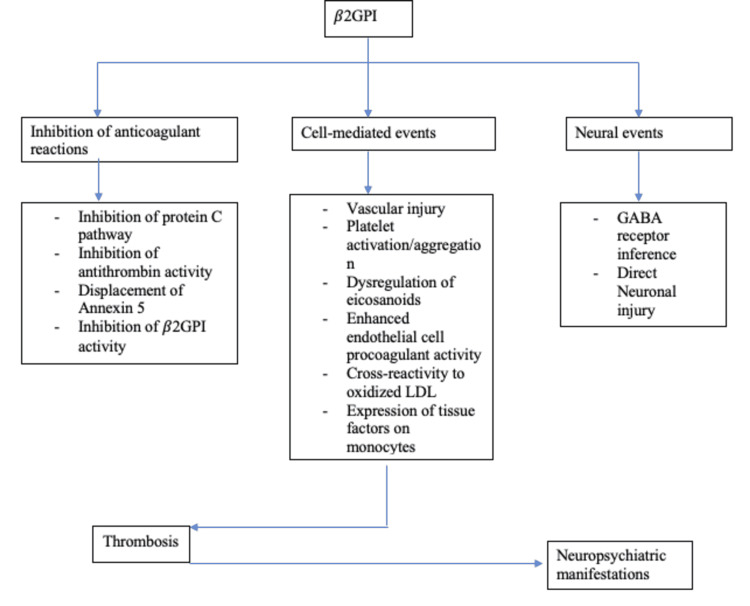
Representing sequence of changes set in motion due to β2GPI activity. β2GPI: beta-2 glycoprotein I, GABA: gamma amino-butyric acid, and LDL: low-density lipoprotein.

The pathophysiology of APS is multifaceted and multilayered, contributed by an interplay of many factors-the predominant and most strongly associated being represented by the presence of beta-2 glycoprotein I (β2GPI) [10]. Several antibodies target this antigen, while anti-domain I antibodies are strongly linked to thrombotic events [11]. Thrombotic events can be arterial or venous and range from mild to catastrophic episodes. The current hypothesis suggests that individuals prone to developing these thrombotic manifestations do so after an external trigger like an infectious prodrome through the mechanism of molecular mimicry [3]. Through a series of conformational changes, the β2GPI becomes enhanced in its antigenic capacity, exposing cryptic epitopes [11]. This immunogenic transformation ("first hit") is enough to create antiphospholipids (aPLs) but not APS warranting a "second hit," which is contributed by several events causing an increase in oxidative stress in endothelial cells like infections, pregnancy, and smoking [12]. Below is a layout representing these events briefly in Figure 1, followed by a representation of changes that are initiated by the activity of β2GPI in Figure 2.

Neurological Pathophysiology

While thrombotic events are responsible for neurological assaults, the role of immunogenic pathophysiology contributes significantly in many cases [13]. It is not clear why the immunogenic mechanisms target neural tissue with more affinity, but data from some animal models suggests that aPL binds to neurons, glial cells, and myelin in feline and murine models [14]. In a study done by Katzav et al. in a murine model, mice developed neurological manifestations like hyperactivity and cognitive impairment without any thrombotic events after intracerebroventricular injection of antiphospholipid antibodies. Furthermore, this study showed that microscopic examination of mice brain tissue revealed mononuclear infiltrates in the choroid plexus and hippocampus without any thrombosis [15]. In another study done by Shoenfeld et al., neuronal-binding antibodies from patients with APS were injected into mice peripherally but passively passed intrathecally and resulted in cognitive impairment tested by the Morris water maze. This study supports that aPL passively entering the brain plays a direct role in neurological pathophysiology [16]. The aPL not only entered the neural tissue passively but also required that the exposure happens for longer times, as demonstrated in a study done by Shrot et al. in a murine model where β2GPI when injected into female mice showed both cognitive and behavioral changes at a higher incidence on week 18 compared to week 6 or week 12 [7]. Moreover, several case reports like the one reported by Horn et al. showed gradual and progressive depression, dementia, and chorea in a 22-year-old woman with lupus anticoagulant positivity [17].

The common point of initiation of the pathophysiology in most of the above-mentioned studies seemed to be the compromise of the blood-brain barrier (BBB), which was demonstrated by the injection of Evans blue (EB) in β2GPI primed mic by Katzav et al. [9]. The mice were sacrificed and residual staining of EB was calculated by EB fluorescence and absorbance spectroscopy on an enzyme-linked immunosorbent assay (ELISA) plate reader. A concentration of EB was seen around the cerebral vasculature, localized in the hippocampus, neocortex, and striatum. Another major finding in this study was in vivo IgG accumulation in cell bodies of the hippocampus stratum radiatum and stratum moleculare (dentate molecular layer). It was also found that interneurons that secrete gamma-aminobutyric acid (GABA) were most affected. This hypothesis was further tested by treating these mice with benzodiazepine and other agents with GABA-mimetic activity, which resulted in the suppression of aggressive behavior and improvement in cognitive testing through the staircase test [9].

In their systemic review, Peluso et al. [18] pointed out the two phases by which aPL positivity results in neuropsychiatric manifestations. The first step is the binding of aPL to brain endothelium, leading to endothelial dysfunction, which results in disruption of the BBB and extravasation of the neurotoxic cytokines and serum proteins, including activated thrombin. In the second step, aPL causes direct damage to the neural tissue. aPL seems to interact with neuronal cell lines with dopaminergic characteristics, modulating the neuronal activity. It also interacts with excitatory pathways through N-methyl-D-alanine (NMDA) glutamate receptor over-activation, as demonstrated in murine models. Immunogold electron microscopy techniques have shown that monoclonal phosphatidyl-serine antibodies react strongly with myelin, resulting in demyelinating disorders as discussed below.

Clinical manifestation of APS

Thrombotic Manifestations

Thrombotic events contribute to a majority of neurological events in cases of APS like cerebrovascular disease, ischemic stroke and transient ischemic attacks (TIA), cerebral venous thrombosis (CVT), Sneddon's syndrome, and other non-inflammatory thrombotic vasculopathy. A detailed discussion of thrombotic manifestations is beyond the scope of this article.

Immunological Manifestations

Cognitive impairment: Cognitive impairment has been reported in multiple case studies and case series of patients presenting with non-criteria APS. Often, cognitive impairment can be subtle or dramatic and catastrophic to patients' caregivers. Often, these patients are treated without an accurate diagnosis. In a review done by Gris and Brenner, the most common cognitive domains involved were complex attention and verbal fluency [19]. Cognitive impairment is present in 19-40% of aPL-positive patients and 42-80% of primary APS patients [20]. In a study done by Erkan et al., 143 APS patients with moderate to high aPL titers were included, and this study found a linear relationship between aPL titers and the severity of cognitive dysfunction [21]. Furthermore, this study showed that the cognitive impairment was of the subcortical type.

In a study conducted by Kao et al., regional blood flow in 22 women with primary APS was done using ^99m^Tc-hexamethyl propylene amine oxime single-photon emission computed tomography. All these women suffered from neuropsychiatric manifestations with normal brain MRI. Hypoperfusion was found in 73% of patients, mainly in the cerebral cortex [22].

Depression: Depression is the most commonly presented neuropsychiatric complaint in APS patients, and it can be present weeks to months before the onset of thrombotic events. It is often not evaluated in the light of autoimmune pathology, and the diagnosis is delayed. The most accepted hypothesis is the effect of aCL on dopaminergic and serotoninergic neurons, thereby causing mood changes. Depression can be the only neuropsychiatric symptom of APS or may be present with acute psychosis or headaches. A case-control study involving patients with depression was done, of which 22 were minor, 23 were major, and 20 were melancholic depressives. A large number of depressed subjects showed aPL positivity indicating some role of the presence of aPL in depression [23].

Dementia: Dementia in young is found to be associated with aCL positivity. Sudden onset dementia can be a presenting feature of APS in an otherwise healthy patient. Acute onset of severe or quickly progressing dementia should prompt evaluation of the autoimmune etiology, especially in the young. In a European study out of 1000 subjects, multi-infarct dementia was shown to affect 2.5% of both primary and secondary APS patients included in the Euro-APS cohort. This type of dementia improves with APS treatment [6]. In the same study, normal individuals with aPL positivity without any symptoms demonstrated inferior cognitive performance on neuropsychiatric evaluation compared to normal individuals with aCL negativity. Case reports showing improvement of dementia in APS patients with immunosuppression exist, but therapeutic guidelines and large multicentric studies are lacking [14]. Sudden onset dementia in young patients without any known cause or family history must be subjected to aPL testing to rule out APS as a cause.

Psychosis: Psychosis is one of the most commonly occurring psychiatric manifestations in APS-delusion, and hallucination is the most commonly occurring psychotic symptom. In a systemic review done by Hallab et al., which included 23 articles, psychosis was presented as paranoia, auditory and visual hallucinations, and occasionally catatonia. The exact pathophysiology of psychosis in APS remains unknown [14]. Psychosis can be a presenting symptom for primary APS, even in younger age groups. A comprehensive study of aPLAbs in blood and CSF from 100 patent with psychosis showed specificity and isotype variability between blood and CSF aPLAbs, indicating a central nervous system (CNS) independent autoimmune process with intrathecal synthesis [16].

Multiple sclerosis-like diseases: A wide spectrum ranging from multiple sclerosis (MS) to MS-like disease to MS-SLE overlap syndrome exists, which is hard to differentiate clinically unless testing for aPL is done. The demographic data between MS and aPL overlaps, which makes it even more difficult to diagnose and differentiate [24]. aPL positivity has been reported in 2-88% of MS patients, with the positivity of aPL increasing with MS relapses in secondary progressive MS compared to relapsing-remitting MS [25]. MS can be distinguished from APS mimicking MS by MRI based on the character of lesions. Lesions due to APS tend to maintain their shape and size on repeat imaging, have lower volumes, are generally subcortical vs periventricular in MS, and do not show the typical ovoid shape or predilection for the corpus callosum. The lesions may affect other regions of the brain like the putamen and do not lessen the brain parenchymal fraction [26].

The pathophysiology through which aPL induces MS-like manifestations has contributed to molecular mimicry with myelin or other CNS proteins. aPL has demonstrated cross-reactivity with myelin, myelin-related protein, and brain phospholipids (cephalin and sphingomyelin) [26]. Sun et al. found that aCL inhibited the proliferation of astrocytes in mouse brains [27].

The systemic review conducted by Ferreira et al. found that high-intensity anticoagulation with warfarin with an international normalized ratio (INR) between three and four was the most effective treatment with low hemorrhagic risk and that primary prevention with anti-aggregation or low-dose oral anticoagulation is probably suboptimal. This review suggested that a trial of oral anticoagulation for six months with a target INR of three to four in patients with persistent aPL might be cost-effective and even beneficial [26].

Neuromyelitis optica spectrum disorders (NMOSDs): NMOSD is rarely present in APS and might sometimes overlap with thrombotic events, masking its manifestations. NMOSD is a group of demyelinating disorders with autoimmune pathology targeting astrocytes that are associated with the presence of anti-aquaporin-4-IgG (AQP4-IgG) [28]. NMOSD can be associated with other autoimmune disorders like rheumatoid arthritis, SLE, and Sjogren's syndrome [29]. aPL antibodies are found in 19-46% of NMOSD patients [30], and plasma exchange is done in these patients, which might show some improvement. Hence, patients with NMOSD must be screened for aPL.

Chorea: Chorea is defined as “an ongoing random-appearing sequence of one or more discrete involuntary movements or movement fragments". It is a rare manifestation of APS with a prevalence of 1.3% in the Euro-phospholipid project group. It occurs due to lesions of the basal ganglia. It is more common in females, with a ratio of 2:1 [6]. Hormonal influences might contribute as a trigger as the incidence of chorea increases in pregnancy or estrogenic therapy, as suggested by Peluso et al. [18], which states that discontinuation of trigger treatments like estroprogestinic therapy and induction of anticoagulant or antiplatelet therapy leads to remission in most cases. In patients whose chorea is contributed additionally by thrombotic events, long-term warfarin treatment is suggested. Traditional neuroleptics like haloperidol have shown results in chorea associated with aPS and SLE, alongside immunosuppression to treat the underlying APS. Renier et al. in their retrospective analysis of 30 patients demonstrated equal efficacy of steroids and neuroleptics when added to previous therapy [31].

The pathogenesis of chorea in APS is largely contributed by a two-phase process where first endothelial dysfunction leads to microthrombi and inflammation in blood vessels, leading to disruption of the BBB, leading to the entry of neural toxins which alter the dopaminergic neural cell lines and also N-Methyl D-Aspartate (NMDA) glutamate receptor over-activation [17].

Hearing loss: Sensorineural hearing loss (SNHL) is a rare occurrence in APS, but it is currently being extensively studied under the broad term “autoimmune SNHL,” or “autoimmune cochleopathy,” or “autoimmune inner ear disease.” It accounts for less than 1% of cases of SNHL. In the absence of trustable markers, autoimmune ear disorders are defined by an appropriate clinical presentation and a positive response to steroid therapy [32]. Several case reports and case series indicate the presence of mild to severe hearing loss secondary to APS.

The pathology of autoimmune inner ear disease (AIED) is contributed by both cell-mediated and humoral immunity. After undefined triggers, the cochlear proteins are exposed to systemic immunity, which then crosses the blood-labyrinthine barrier to reach the endolymphatic sac, which is the major modulator of ear immunity. Chronic exposure to systemic immune mechanisms leads to the destruction of sensory and supporting cells in the cochlea. In murine models, circulating antibodies against collagen type ii, type ix, Raf-1 protein, major peripheral myelin protein P0, B-actin, cochlin, choline transporter-like protein 2 (CTL2), cell density enhanced protein tyrosine phosphatase-1, and connexin 26 have been described to cause AIED [32].

MRI changes in APS: More often than not, MRIs done in asymptomatic APS patients are normal. In an observational study conducted by Wan et al. [33], 44 primary APS (PAPS) patients were enrolled, including 24 aPL carriers and 23 healthy controls without any history of neuropsychiatric manifestations. About 38 (55.88%) out of the 44 patients with PAPS showed some persistent abnormal MRI findings, of which lacunes were the most frequent (45.59%) followed by white matter hyperintensities (29.41%). Furthermore, this study showed that female gender and thrombocytopenia were significant independent risk factors for abnormal MRI. This study recommended that the high prevalence of abnormalities in MRI emphasized on attention to silent cerebral lesions in patients with persistent aPL positivity. Such patients are highly at risk of thrombotic events and might benefit from primary thromboprophylaxis with a low dose of aspirin, although definite data on the risks/benefits ratio is lacking.

Several other neuropsychiatric manifestations occur in APS, for instance, transverse myelitis, myelopathies, and movement disorders like ballismus, dyskinesia, parkinsonism, and other cerebellar ataxia [34].

Therapeutic options

Treatment in APS patients is decided on a case-to-case basis depending on the risk-benefit ratio of the therapy opted for. According to the European Alliance of Associations for Rheumatology (EULAR) recommendations, primary prophylaxis with low-dose aspirin (LDA) in aPL-positive patients who are asymptomatic showed a reduced risk of the first thrombosis by at least 50%. In patients with APS and first venous thrombosis treated with unfractionated heparin or low molecular weight heparin, a secondary thromboprophylaxis with vitamin K antagonist (VKA) is recommended [2].

The main challenge of treating APS with non-thrombotic neuropsychiatric manifestations is the low certainty of evidence. Many cases reporting the use of therapeutic intravenous immunoglobulin (IVIg) or high-dose steroid injections or other immunosuppressive agents in cases of acute psychiatric events have been documented without any organized stratification of data.

In the systematic review conducted by Hallab et al. that included 23 papers, 17 papers reported the use of antipsychotics, and five of those patients were given a combination of two antipsychotics. GABA mimetics were found to be the most effective treatment option for psychotic symptoms by acting on the mesolimbic pathway dopaminergic neurons. Dumitrescu et al. did not use any GABA mimetic agents and found no improvement in psychotic symptoms [35]. Eight studies showed the use of antidepressants like selective serotonin reuptake inhibitors (SSRIs), while four studies reported the use of anxiolytics and sedatives mainly prescribed for convulsions. The most commonly used antiepileptic was phenytoin, while levetiracetam was avoided in lieu of associated psychosis in the majority of cases. These different classes of medication were combined in at least nine studies [36]. The prognosis was favorable in 19 cases and poor in two cases, while two previously treated patients relapsed after discontinuing aspirin.

In all of the studies, the underlying APS was treated with appropriate medication considering pregnancy, age of onset, and severity of symptoms.

## Conclusions

APS commonly presents with neurological impairment secondary to thrombotic events, however increasingly published case reports and case series suggest psychiatric manifestations as presenting symptoms of APS. Even as cases of non-thrombotic neuropsychiatric manifestations of APS are on the rise, concrete data indicating its incidence, outcome, and treatment strategies are lacking. The treatment plan as of now varies amongst physicians and is decided in conversion between a neurologist and a rheumatologist, low-dose aspirin is the most commonly administered prophylactic treatment. The shortcomings of these articles are the unavailability of data from large multicentric trials and standard treatment criteria. Further research in this direction is recommended.

## References

[1] Miyakis S, Lockshin MD, Atsumi T (2006). International consensus statement on an update of the classification criteria for definite antiphospholipid syndrome (APS). J Thromb Haemost.

[2] Durcan L (2016). Epidemiology of the antiphospholipid syndrome. Handbook of Systemic Autoimmune Diseases, Antiphospholipid Syndrome in Systemic Autoimmune Diseases, Volume 12.

[3] Schreiber K, Sciascia S, de Groot PG (2018). Antiphospholipid syndrome. Nat Rev Dis Primers.

[4] Wilson WA, Gharavi AE, Koike T (1999). International consensus statement on preliminary classification criteria for the antiphospholipid syndrome: report of an international workshop. Arthritis Rheum.

[5] Cervera R, Piette JC, Font J (2002). Antiphospholipid syndrome: clinical and immunologic manifestations and patterns of disease expression in a cohort of 1,000 patients. Arthritis Rheum.

[6] Sorice M, Longo A, Capozzi A (2007). Anti-beta2-glycoprotein I antibodies induce monocyte release of tumor necrosis factor alpha and tissue factor by signal transduction pathways involving lipid rafts. Arthritis Rheum.

[7] Shrot S, Katzav A, Korczyn AD (2002). Behavioral and cognitive deficits occur only after prolonged exposure of mice to antiphospholipid antibodies. Lupus.

[8] Frauenknecht K, Katzav A, Weiss Lavi R, Sabag A, Otten S, Chapman J, Sommer CJ (2015). Mice with experimental antiphospholipid syndrome display hippocampal dysfunction and a reduction of dendritic complexity in hippocampal CA1 neurones. Neuropathol Appl Neurobiol.

[9] Katzav A, Menachem A, Maggio N, Pollak L, Pick CG, Chapman J (2014). IgG accumulates in inhibitory hippocampal neurons of experimental antiphospholipid syndrome. J Autoimmun.

[10] de Laat B, Pengo V, Pabinger I (2009). The association between circulating antibodies against domain I of beta2-glycoprotein I and thrombosis: an international multicenter study. J Thromb Haemost.

[11] Ioannou Y, Zhang JY, Qi M (2011). Novel assays of thrombogenic pathogenicity in the antiphospholipid syndrome based on the detection of molecular oxidative modification of the major autoantigen beta2-glycoprotein I. Arthritis Rheum.

[12] Agar C, van Os GM, Mörgelin M (2010). Beta2-glycoprotein I can exist in 2 conformations: implications for our understanding of the antiphospholipid syndrome. Blood.

[13] Erkan D, Lockshin MD (2004). What is antiphospholipid syndrome?. Curr Rheumatol Rep.

[14] Hallab A, Naveed S, Altibi A (2018). Association of psychosis with antiphospholipid antibody syndrome: a systematic review of clinical studies. Gen Hosp Psychiatry.

[15] Katzav A, Ben-Ziv T, Blank M, Pick CG, Shoenfeld Y, Chapman J (2014). Antibody-specific behavioral effects: intracerebroventricular injection of antiphospholipid antibodies induces hyperactive behavior while anti-ribosomal-P antibodies induces depression and smell deficits in mice. J Neuroimmunol.

[16] Shoenfeld Y, Nahum A, Korczyn AD (2003). Neuronal-binding antibodies from patients with antiphospholipid syndrome induce cognitive deficits following intrathecal passive transfer. Lupus.

[17] Van Horn G, Arnett FC, Dimachkie MM (1996). Reversible dementia and chorea in a young woman with the lupus anticoagulant. Neurology.

[18] Peluso S, Antenora A, De Rosa A, Roca A, Maddaluno G, Brescia Morra V, De Michele G (2012). Antiphospholipid-related chorea. Front Neurol.

[19] Gris JC, Brenner B (2013). Antiphospholipid antibodies: neuropsychiatric presentations. Semin Thromb Hemost.

[20] Weizman R, Paz L, Peter Y, Toren P, Pick CG (2001). Behavioral effects of agents active at the gamma-aminobutyric acid receptor complex in the staircase paradigm. Brain Res.

[21] Erkan D, Barbhaiya M, George D, Sammaritano L, Lockshin M (2010). Moderate versus high-titer persistently anticardiolipin antibody-positive patients: are they clinically different and does high-titer anti-β2-glycoprotein-I antibody positivity offer additional predictive information?. Lupus.

[22] Kao CH, Lan JL, Hsieh JF, Ho YJ, ChangLai SP, Lee JK, Ding HJ (1999). Evaluation of regional cerebral blood flow with 99mTc-HMPAO in primary antiphospholipid antibody syndrome. J Nucl Med.

[23] Maes M, Meltzer H, Jacobs J, Suy E, Calabrese J, Minner B, Raus J (1993). Autoimmunity in depression: increased antiphospholipid autoantibodies. Acta Psychiatr Scand.

[24] Cuadrado MJ, Khamashta MA, Ballesteros A, Godfrey T, Simon MJ, Hughes GR (2000). Can neurologic manifestations of Hughes (antiphospholipid) syndrome be distinguished from multiple sclerosis? Analysis of 27 patients and review of the literature. Medicine.

[25] D'Angelo C, Franch O, Fernández-Paredes L (2019). Antiphospholipid antibodies overlapping in isolated neurological syndrome and multiple sclerosis: neurobiological insights and diagnostic challenges. Front Cell Neurosci.

[26] Ferreira S, D'Cruz DP, Hughes GR (2005). Multiple sclerosis, neuropsychiatric lupus and antiphospholipid syndrome: where do we stand?. Rheumatology.

[27] Sun KH, Liu WT, Tsai CY, Liao TJ, Lin WM, Yu CL (1992). Inhibition of astrocyte proliferation and binding to brain tissue of anticardiolipin antibodies purified from lupus serum. Ann Rheum Dis.

[28] Jarius S, Paul F, Weinshenker BG, Levy M, Kim HJ, Wildemann B (2020). Neuromyelitis optica. Nat Rev Dis Primers.

[29] Iyer A, Elsone L, Appleton R, Jacob A (2014). A review of the current literature and a guide to the early diagnosis of autoimmune disorders associated with neuromyelitis optica. Autoimmunity.

[30] Lee EJ, Lim YM, Kim SY (2019). The clinical and prognostic value of antinuclear antibodies in NMO-IgG seropositive neuromyelitis optica spectrum disorder. J Neuroimmunol.

[31] Reiner P, Galanaud D, Leroux G (2011). Long-term outcome of 32 patients with chorea and systemic lupus erythematosus or antiphospholipid antibodies. Mov Disord.

[32] Mijovic T, Zeitouni A, Colmegna I (2013). Autoimmune sensorineural hearing loss: the otology-rheumatology interface. Rheumatology.

[33] Wan L, Liu T, Chen T (2022). The high prevalence of abnormal magnetic resonance imaging findings in non-neuropsychiatric patients with persistently positive anti-phospholipid antibodies. Rheumatology.

[34] Huang YC, Lyu RK, Chen ST, Chu YC, Wu YR (2008). Parkinsonism in a patient with antiphospholipid syndrome-case report and literature review. J Neurol Sci.

[35] Dumitrescu L, Nicolau A, Popescu BO (2013). Kaleidoscopic nervous system involvement in the setting of a chronic multisystem dysimmune disorder: report of a remarkable case. Rom J Neurol.

[36] Fleetwood T, Cantello R, Comi C (2018). Antiphospholipid syndrome and the neurologist: from pathogenesis to therapy. Front Neurol.

